# Supplementary material for: Outbreak of acute gastroenteritis associated with drinking water in rural Kazakhstan: A matched case-control study
Source: PLOS Glob Public Health. 2022 Dec 9;2(12):e0001075. doi: 10.1371/journal.pgph.0001075 (PMC10021418; doi:10.1371/journal.pgph.0001075)
Supplement: Attachment — Submitted filename: Response to Reviewers.docx [file pgph.0001075.s001.docx]

**Author response to editor comments:**

1 Please review your reference list to ensure that it is complete and correct. If you have cited papers that have been retracted, please include the rationale for doing so in the manuscript text, or remove these references and replace them with relevant current references. Any changes to the reference list should be mentioned in the rebuttal letter that accompanies your revised manuscript. If you need to cite a retracted article, indicate the article’s retracted status in the References list and also include a citation and full reference for the retraction notice.

*Author response: We have checked this.*

2. Please insert an Ethics Statement at the beginning of your Methods section, under a subheading 'Ethics Statement'. It must include:

a) The name(s) of the Institutional Review Board(s) or Ethics Committee(s)

b) The approval number(s), or a statement that approval was granted by the named board(s)

c) (for human participants/donors) - A statement that formal consent was obtained (must state whether verbal/written) OR the reason consent was not obtained (e.g. anonymity). NOTE: If child participants, the statement must declare that formal consent was obtained from the parent/guardian.

*Author response: We have added this. We also made it clear that written consent was obtained and that no interviews were conducted with children, information on children activities were obtained from parents.*

3. Please amend your detailed Financial Disclosure statement. This is published with the article. It must therefore be completed in full sentences and contain the exact wording you wish to be published.

a. State the initials, alongside each funding source, of each author to receive each grant.
b. State what role the funders took in the study. If the funders had no role in your study, please state: “The funders had no role in study design, data collection and analysis, decision to publish, or preparation of the manuscript.”

If you did not receive any funding for this study, please simply state: “The authors received no specific funding for this work.”

*Author response: We amended it.*

4. We noticed that you used “data not shown” in the manuscript. We do not allow these references, as the PLOS data access policy requires that all data be either published with the manuscript or made available in a publicly accessible database. Please amend the supplementary material to include the referenced data or remove the references.

*Author response: We added the result to Table 1 and removed “data not shown”.*

5. Please provide separate figure files in .tif or .eps format only and remove any figures embedded in your manuscript file. Please also ensure that all files are under our size limit of 10MB.

*Author response: We removed the figure and attached the .eps or .tif*

6. In the online submission form, you indicated that "An anonymized limited data set can be made available upon request". All PLOS journals now require all data underlying the findings described in their manuscript to be freely available to other researchers, either 1. In a public repository, 2. Within the manuscript itself, or 3. Uploaded as supplementary information.
This policy applies to all data except where public deposition would breach compliance with the protocol approved by your research ethics board. If your data cannot be made publicly available for ethical or legal reasons (e.g., public availability would compromise patient privacy), please explain your reasons by return email and your exemption request will be escalated to the editor for approval. Your exemption request will be handled independently and will not hold up the peer review process, but will need to be resolved should your manuscript be accepted for publication. One of the Editorial team will then be in touch if there are any issues.

*Author response: We added the data to a public repository with link.*

**Reviewer #1: Author response**

1. Abstract
Clarify whether these suspected cases were included in the case group.

*Author response: We agree this can be confusing, we clarified that all cases are suspected cases because we did not know the etiology of disease.*

2. Metodology
I suggest characterizing the control together with the case group in table 1 in study design;

*Author response: We added information* group *for the control group.*

I suggest trying to better detail the frequency of exposure of the two groups (case and control) mainly to the use of unboiled tap water in data collection;

*Author response: We agree that assessing frequency of exposure would be the best approach. Unfortunately, that information is not available. However, our analysis does control for having drank from other sources (water dispensers and bottled beverages).*

I suggest adding the graphs that demonstrate the statistical analyzes in data analysis;

*Author response: We agree that assessing frequency of exposure would be the best approach. Unfortunately, that information was not available.*

I suggest explaining why only 12 samples were collected of fresh stool specimens in laboratory testing.

*Author response: All cases were asked to provide stool, but only 12 consented. We added this information to methods and also the discussion does include that this is a limitation of our analysis. Laboratory testing does provide additional support to the conclusions, but even without laboratory data, our conclusions would be the same based on strong evidence from non-lab epidemiological analysis*

3. Discussion
I suggest deepening the discussion by comparing the results obtained in this research with other studies already published on the subject.

*Author response: We added comparisons to the discussion section.*

**Reviewer #2: Author response**

Two points drew attention: The presentation of the data in greater detail and that the investigation is an activity, not being considered research, implying that it does not need the authorization of the ethics committee, only from the Ministry of Health.

*Author response: Yes, you are correct this is an outbreak response activity and not research. This was reviewed by U.S. Centers for Disease Control and Prevention and the Ministry of Health which received approval as non-research activity. We clarified this with the statement – “This investigation was reviewed and received approval as a non-human activity by U.S. Centers for Disease Control and Prevention under 45 CFR 46.102(l)(2 and the Kazakhstan Ministry of Health Committee No. 24-03-21/2703 dated June 03, 2021.” We have also put the data in a public repository.*

**Reviewer #3: Author response**

1- In the methodology, better specify the exposure, graduating the degree of exposure (for example: did you use inadequate water once or is this routine consumption?), in order to make the group more homogeneous.

*Author response: We agree that assessing frequency of exposure would be the best approach. Unfortunately, that information is not available. However, our analysis does control for having drank from other sources (water dispensers and bottled beverages).*

2- Increase the discussion with other articles in the literature, especially those that include data from nearby populations that consume water with similar treatment.

*Author response: We added comparisons to the discussion section.*

3- The results are well described, but I suggest including graphs to illustrate the results in a more didactic way.

*Author response: We have included a map that demonstrates how cases are clustered in the section of town and a histogram.*

I suggest adding the overview of gastroenteritis on site, pointing out in one line, no most, the importance of studying the topic. The first line conveys the idea of being the objective of the study, followed by the methodology, results and conclusion that, if they are better "stitched", will convey a better idea to the reader.

*Author response: This is not a study. We are instead providing results of an outbreak investigation, which generally tend to follow a more narrative timeline based format rather than the traditional IMRAD format.*

Line 67: Date in abstract (line 34) is different in study design.

*Author response: Thank for such attention to detail in your review. The abstract includes dates patients were actually interviewed and the methods section includes the entire span the team was out conducting the outbreak investigation (which included site analysis). While the dates are correct in both instances, we agree that it can be confusing so we updated the abstract to span the entire outbreak investigation rather than limiting it to only days of interviews.*

- Line 68 to 69: is the health care unit the only one found in the village? I suggest adding more details about it.

*Author response: We added that all facilities were included.*

- Line 71: "The list of residents", potential controls were identified in what way (neighborhood, door-to-door, friends, phone number...). I propose to researchers to better clarify the approach used to identify control houses in order to avoid group selection bias.

*Author response: We added that this is a resident database which includes demographic information on all people who resides in the village.*

- Line 72 to 73: I recommend bringing in table 1 the existing pairing between control and cases. The objective is to avoid the situation of confusion and increase the efficiency of the study.

*Author response: We added this.*
